# OXA-66 structure and oligomerisation of OXA*Ab* enzymes

**DOI:** 10.1099/acmi.0.000412

**Published:** 2022-10-03

**Authors:** Yuiko Takebayashi, Sara R. Henderson, Dimitri Y. Chirgadze, Philip J. Warburton, Benjamin A. Evans

**Affiliations:** ^1^​ Department of Biomedical and Forensic Science, Anglia Ruskin University, Cambridge, UK; ^2^​ School of Cellular and Molecular Medicine, University of Bristol, Bristol, UK; ^3^​ Norwich Medical School, University of East Anglia, Norwich, UK; ^4^​ Institute of Microbiology and Infection, College of Medical and Dental Sciences, University of Birmingham, Birmingham, UK; ^5^​ Department of Biochemistry, University of Cambridge, Cambridge, UK; ^6^​ School of Biomedical Sciences, Faculty of Health, University of Plymouth, Plymouth, UK

**Keywords:** acinetobacter, antibiotic resistance, beta-lactamase, carbapenemase

## Abstract

The OXA β-lactamases are responsible for hydrolysing β-lactam antibiotics and contribute to the multidrug-resistant phenotype of several major human pathogens. The OXA*Ab* enzymes are intrinsic to *Acinetobacter baumannii* and can confer resistance to carbapenem antibiotics. Here we determined the structure of the most prevalent OXA*Ab* enzyme, OXA-66. The structure of OXA-66 was solved at a resolution of 2.1 Å and found to be very similar to the structure of OXA-51, the only other OXA*Ab* enzyme that has had its structure solved. Our data contained one molecule per asymmetric unit, and analysis of positions responsible for dimer formation in other OXA enzymes suggest that OXA-66 likely exists as a monomer.

## Data summary

The crystal structure of OXA-66 has been deposited in the Protein Data Bank (PDB) with the PDB ID: 6T1H. The coordinate files, diffraction data and validation report can be downloaded from the PDB, DOI: 10.2210/pdb6t1h/pdb.

## Introduction

The OXA-type β-lactamases are enzymes that hydrolyse the β-lactam antibiotics. They are commonly found in Gram-negative bacteria that cause serious infections in humans, including *Pseudomonas aeruginosa *, *Escherichia coli *, *Klebsiella pneumoniae *, and *Acinetobacter baumannii* [1]. While some groups of OXA enzymes, such as OXA-48, have been mobilised on plasmids and become globally spread in highly successful multidrug-resistant bacterial lineages, others represent intrinsic enzymes belonging to specific bacterial species and are encoded on the chromosome. One such group are the OXA*Ab* enzymes that are intrinsic to *A. baumannii *. It has recently been shown that these intrinsic enzymes are capable of conferring resistance to the carbapenem antibiotics [2], and it is therefore important to understand the molecular mechanisms behind this. To date, only the structure of the OXA*Ab* enzyme OXA-51 has been solved [3]. However, by far the most prevalent OXA*Ab* enzyme is OXA-66 due to its association with the predominant *A. baumannii* Global Clone 2. We therefore sought to determine the structure of OXA-66 and compare it to OXA-51.

Although the OXA-type enzymes have been reported to have a high degree of structural similarity despite their divergence in sequence, there have been contradictory reports suggesting that the oligomerisation of these enzymes may not be universal. For example, some enzymes have been reported to be monomers such as OXA-1 where an elongated Ω-loop is postulated to inhibit the process of dimerization [4], or OXA-25 where an inward bend of the β−3 strand is suggested to destabilise dimerization [5]. In contrast, OXA-48 was determined to be a dimer with a series of salt bridges stabilising an alternative dimeric interface [6, 7]. A third group, including OXA-10 and OXA-14, are only dimeric in the presence of metal ions [8]. Furthermore, for those enzymes that require metal ions for dimerization, it has been determined that the dimer form is more active than the monomeric form, but dimerization is destabilised by the presence of the substrate [8]. Currently there has been no report of any OXA-51-like enzymes having their oligomerisation state confirmed, with only the N-terminal fragment of OXA-58 from *A. baumannii* being classed and found to be a monomer, although there is no evidence of the oligomerisation state of the full length enzyme [9].

Here we present a new structure of the most prevalent OXA*Ab* enzyme, OXA-66, and compare it to the previously solved structures of OXA-51. Additionally we propose, based on similarity and structural models, that this sub-group of enzymes is of a monomeric nature.

## Methods

### Macromolecule production

For crystal structure determination, OXA-66 was amplified by PCR without its signal peptide, using the OXA-66-BamHI forward and reverse primers (Table 1). The signal peptide was predicted using the SignalP 4.1 Server [10, 11]. The insert was subcloned into pGEM-T Easy (Promega, United Kingdom) and confirmed by sequencing with the universal T7 Promoter primer. For expression of OXA-66 fused with a glutathione S-transferase (GST) tag, the insert was digested with BamHI, ligated into the protein expression vector pGEX-6P-1 (GE Healthcare, United Kingdom) and transformed into *E. coli* DH5α. Transformants were selected with ampicillin (100 mg l^−1^) and confirmed by PCR using pGEX Sequencing Primers (GE Healthcare Life Sciences, United Kingdom). The recombinant plasmid was transformed into *E. coli* BL21 (DE3) (Bioline, United Kingdom) following manufacturer guidelines.

**Table 1.** Macromolecule production information

Source organism	* Acinetobacter baumannii *
OXA-66-BamHI Forward primer	AAAGGATCCATGAATCCAAATCACAGC
OXA-66-BamHI Reverse primer	AAAGGATCCCTATAAAATACCTAATTGTTC
Cloning vector	pGEM-T Easy
Expression vector	pGEX-6P-1
Expression host	* E. coli* BL21 (DE3)

BL21 (DE3) pGEX-6P-1 OXA-66 was grown at 37 °C to an OD600 of 0.8–1.0 in LB broth before inducing with a final IPTG concentration of 0.1 mM for 6 h. A total of 11.5 g of cell pellet was yielded for purification. OXA-66 was purified according to the column chromatography method outlined by the GST-Bind Kit (Novagen, United Kingdom), up to the first flow through fraction collection. Subsequently, the manufacturer protocol for PreScission Protease cleavage of GST-tagged protein bound to the column (GE Healthcare, United Kingdom) was followed to remove the GST-tag and elute OXA-66. Homogeneity of the purified protein was confirmed by running the eluted sample on a 10 % Bis-Tris SDS-PAGE gel (Life Technologies, United Kingdom). The purified protein appeared as a single band (data not shown).

### Crystallization

Octahedron crystals were grown under conditions described in Table 2. Crystals used for X-ray diffraction experiments were harvested at 8 days in the precipitant supplemented with 26 % (v/v) ethylene glycol with liquid nitrogen.

**Table 2.** Crystallization conditions

Method	Sitting drop
Plate type	MRC crystallisation plates (SWISSCI, Wokingham, UK)
Temperature (K)	292
Protein concentration (mg ml-1)	22
Buffer composition of protein solution	20 mM HEPES, 50 mM NaCl, pH 7.5
Composition of reservoir solution	0.1 M MES pH 6.5, 22.85 % (v/v) PEG MME 550, 10 mM ZnSO_4_
Volume and ratio of drop	400 nl 1 : 1
Volume of reservoir (µl)	70

### Data collection and processing

The X-ray diffraction dataset was collected a wavelength of 1.5418 Å using a copper rotating anode X-ray diffraction system equipped with confocal mirror monochromator, a kappa geometry goniometer, and Platinum 135 CCD detector (PROTEUM X8, Bruker AXS, Ltd) at a temperature of 100K (Oxford Cryosystems, Ltd). The exposure time was set to 60 s for a single phi-oscillation image of 1 degree, and the total of 1398 oscillation images were collected in eight different kappa geometry orientations. The dataset was indexed, scaled and merged using PROTEUM2 data processing software [12]. The resultant data-collection statistics are summarized in Table 3.

**Table 3.** Data collection and processing

Diffraction source	In-house, Copper rotating anode.
Wavelength (Å)	1.5418
Temperature (K)	100
Detector	Platinum 135 CCD (PROTEUM X8, Bruker AXS, Ltd)
Rotation range per image (°)	1
Total rotation range (°)	1398
Exposure time per image (s)	60
Space group	*P*4_3_22
*a*, *b*, *c* (Å)	87.54, 87.54, 90.12
α, β, γ (°)	90, 90, 90
Resolution range (Å)	45.06–2.10* (2.20–2.10)^†^
No. of unique reflections	21 092 (2689)
Completeness (%)	100 (100)
Redundancy	75.4 (45.7)
<I/σ(*I*)>	28.17 (3.36)
*R*_p.i.m._(%)^‡^	1.9 (11.2)
Overall *B* factor from Wilson plot (Å^2^)	29.1
Number of molecules per asymmetric unit	1
Matthews Coefficient (V_m_)	2.82

*The resolution cut-off criteria was based on the data strength, i.e. only the resolution shells with <I/σ(I)> > 3 were included.

†Values for the outer resolution shell are given in parentheses.

‡*R*_p.i.m_. = (**Σ *_hkl_*** [1/(N-1)]^1/2^ **Σ *_i_*** | *I_i_* (hkl) - *I_mean_* (hkl)|) / **Σ *_hkl_* Σ *_i_*** *I_i_* (hkl), where N is redundancy (p.i.m. – precision-indicating R-factor).

### Structure solution and refinement

The OXA-66 crystal structure was solved by the Molecular Replacement (MR) method. The crystal structure of OXA-51 beta-lactamase (PDB-ID: 4ZDX) was used as the MR search probe. The sequence identity between the search probe and OXA-66 is 97 %. All MR calculations were performed in PHASER, part of the PHENIX crystallographic software suite [13, 14]. The obtained model was subjected to several rounds of alternating manual rebuilding performed in the molecular graphics software suite COOT and crystallographic refinement calculations in PHENIX crystallographic software suite [13, 15]. Final refinement and validation statistics are summarized in Table 4. All molecular graphics and structural analyses were carried out within the CCP4MG and CCP4 suite [16].

**Table 4.** Structure solution and refinement

Resolution range (Å)	43.7720–2.1000 (2.1526–2.1001)^*^
Completeness (%)	99.5 (99.5)
σ cutoff	*F* >1.43σ (*F*)
No. of reflections, working set	18 970 (1316)
No. of reflections, test set	1990 (140)
Final *R*_cryst_	0.188 (0.2470)
Final *R*_free_	0.224 (0.2954)
No. of non-H atoms	
Protein	1 917
Ligand	0
Solvent	229
Total	2 150
R.m.s. deviations	
Bonds (Å)	0.007
Angles (°)	0.843
Average *B* factors (Å^2^)	
Protein	27.2
Ligand	29.1
Ramachandran plot	
Most favoured (%)	98.33
Allowed (%)	1.67

*Values for the outer resolution shell are given in parentheses.

## Results and discussion

**Fig. 1.**
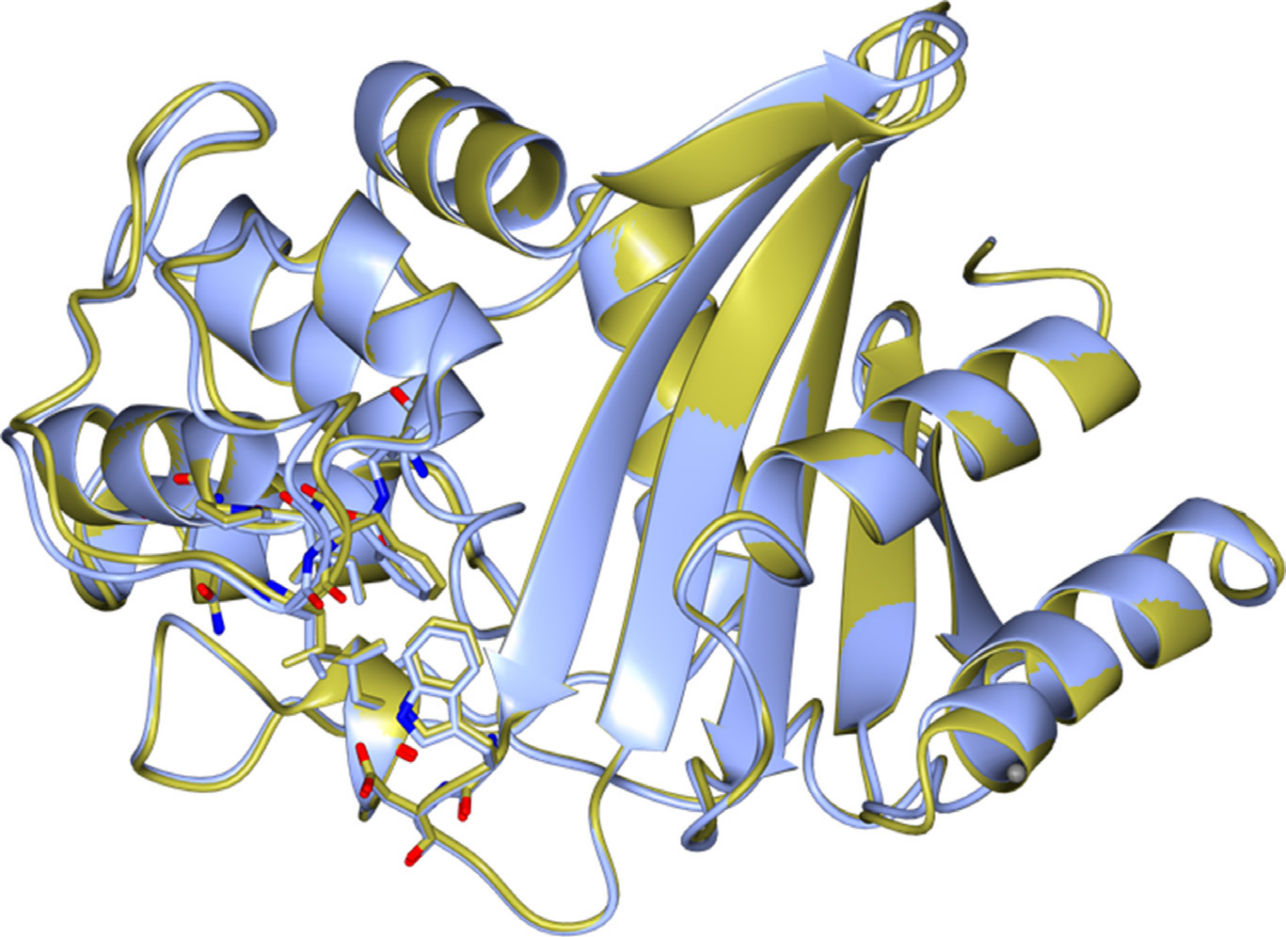
Structural alignment of our OXA-66 structure (blue) with the apo OXA-51 structure 4ZDX (gold), displaying the key active site residues.

We present here the crystal structure of the most prevalent member of the OXA*Ab* group, OXA-66, at a maximum resolution of 2.1 Å, solved by molecular replacement using the OXA-51 structure (PDB ID: 4ZDX) as the molecular replacement model with an amino acid sequence identity of 97 %. As expected, there is as high degree of structural similarity between these enzymes, with an R.M.S.D value of 0.48 Å on 239 c-alpha atoms with the 4ZDX apo structure of OXA-51. OXA-66 differs from OXA-51 by six amino acids – T5A, E36V, V48A, Q107K, P194Q, D225N [17], but this variation in the observed sequences does not appear to affect the main chain or give rise to any significantly altered charge interactions in or near the active site (Fig. 1). This is consistent with the very similar levels of phenotypic resistance to the carbapenem antibiotics that these two enzymes have been shown to confer [2].

Similar to the active site in the Apo OXA-51 structures (5ZKH and 4ZDX), we observe that the tryptophan at position 222 lies in a conformation disfavouring the substrate from binding in the active site (fig. S1, available in the online version of this article) [3, 18]. However, an alternative conformation is observed in the Doripenem bound OXA-51 structure (5L2F) whereby the tryptophan adopts a position pointing away from the substrate coordinating with a water molecule to the substrate to enable binding [18]. This has previously been suggested as an explanation into the weak activity of this sub-class of enzymes [3].

**Fig. 2.**
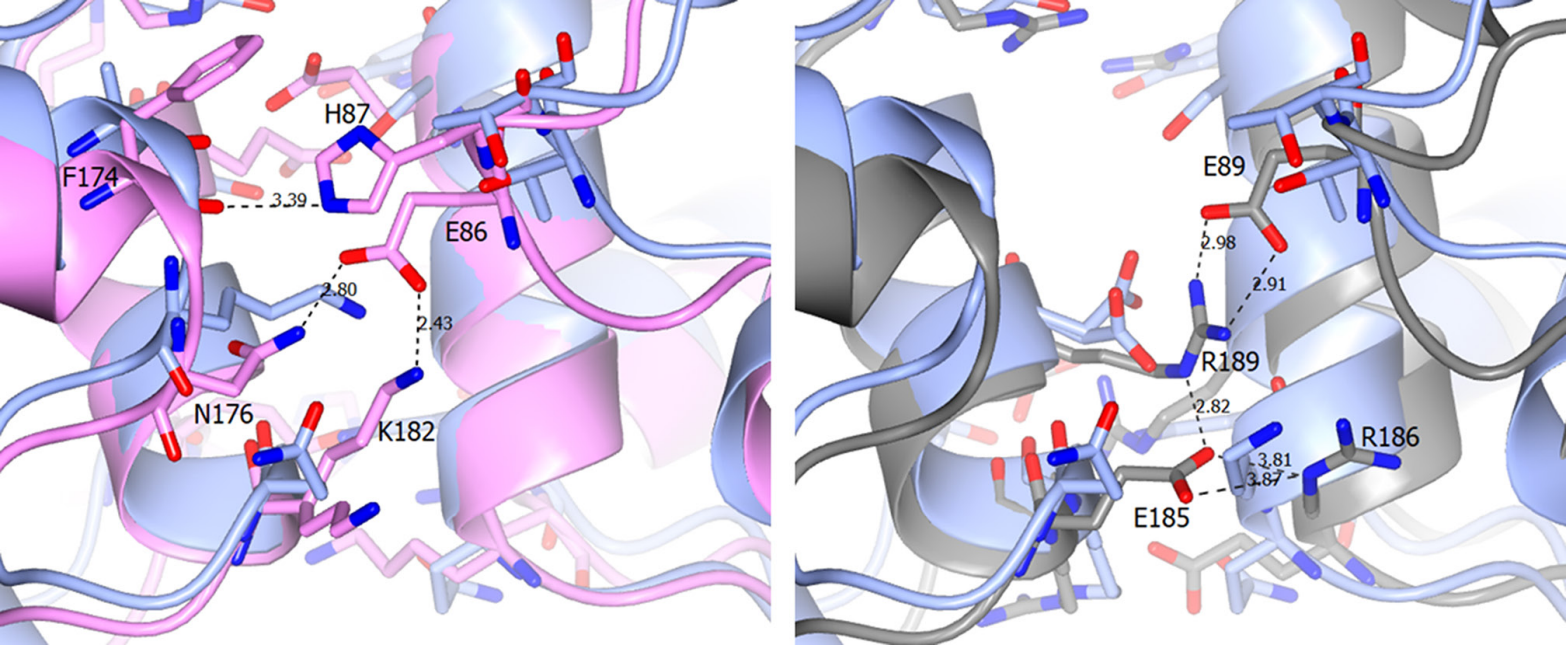
View of part of the dimer interface of (left) OXA-13 (pink, PDB ID: 1H8Z) and (right) OXA-48 (grey, PDB ID: 5DTK) aligned with two copies of OXA-66 (blue). Ionic interactions and bonding distances are represented. No ionic interactions were found at the OXA-66 interface.

One of the interesting features of the OXA enzymes is the varying reports of these highly similar enzymes being either monomeric or dimeric, although at present no OXA*Ab* enzyme has currently been classified as either a dimer or monomer. We therefore compared our OXA-66 structure with confirmed monomeric and dimeric enzymes to determine the possible oligomeric state. Three schemes of salt bridges have been identified as being important for dimerization previously with several key residues identified [7, 19, 20]. These three systems have been identified in OXA-10, OXA-13 and OXA-48. Comparing the sequence of OXA-66 with each of these systems we observe the likely absence of most or all the salt bridges in OXA-66. Both OXA-13 and OXA-48 form native dimers utilising 5–6 salt bridges on an interface distant to the active site [6, 7, 19]. Comparison to the OXA-66 sequence suggests that these salt bridges are likely to be abolished or significantly weakened throughout, suggesting OXA-66 may be monomeric. For example, the glutamic acid at position 86 in OXA-13 forms salt bridges to both Lys182 and Asp176, and the equivalent Glu89 in OXA-48 forms interactions with Arg189. However, in the OXA-66 structure a threonine is present at this position in 3D-space which is much shorter and cannot reach across the gap efficiently to form a salt bridge, especially as the alternative position for Arg189 is a negatively charged aspartic acid residue hence there is likely repulsion at this site (Fig. 2). Likewise, any conserved charges present around this region do not appear to be in a position that would enable salt bridge formation.

**Fig. 3.**
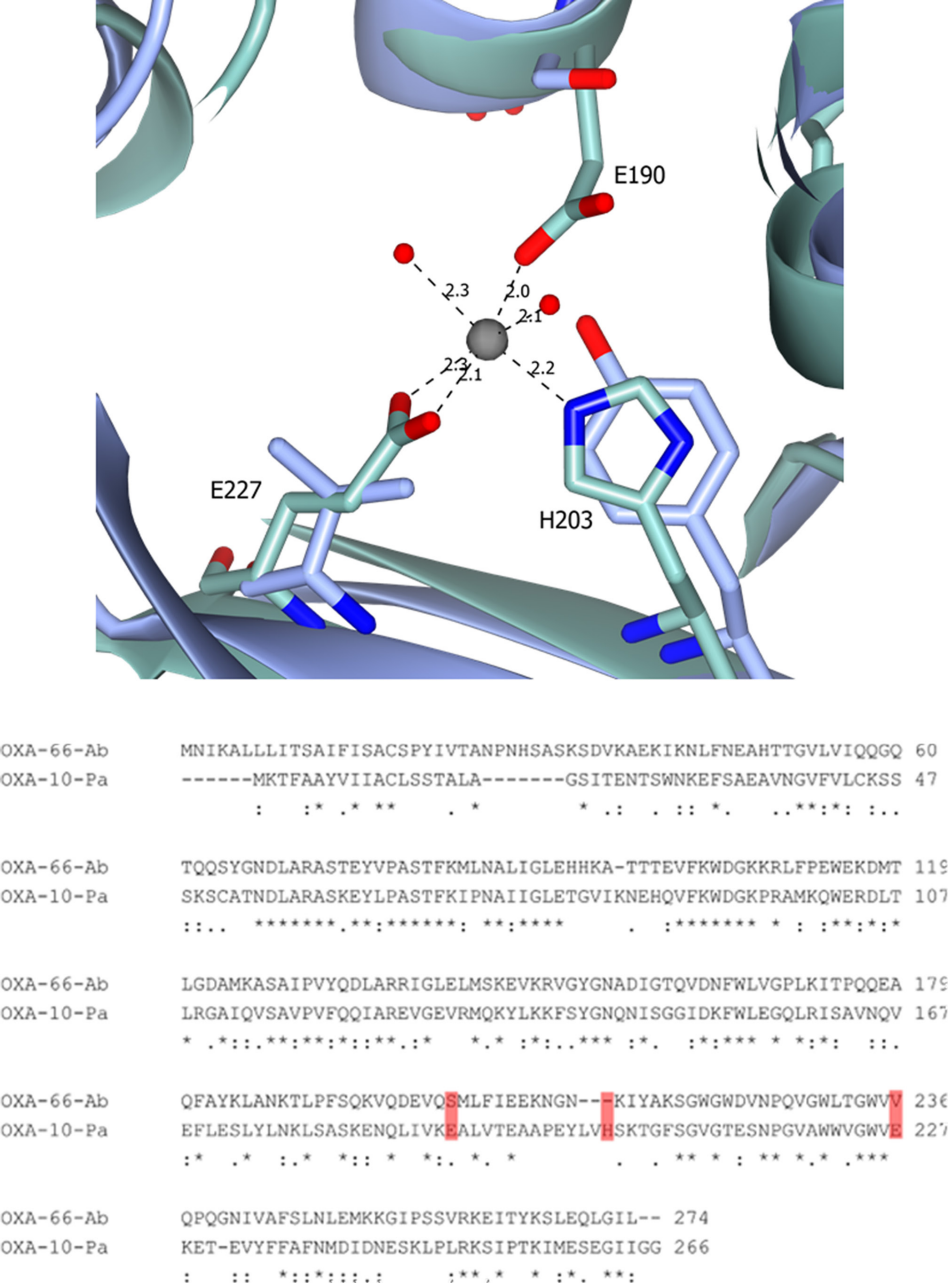
Top: Metal coordination forming dimer interaction in OXA-10 (green), overlaid with two copies of OXA-66 (blue), where no salt bridge or metal co-ordination is observed in comparison to the OXA-10 metal coordination. Numbering refers to OXA-10 structure. Bottom: Sequence alignment of OXA-66-Ab with OXA-10-Pa demonstrating the absence of charge conservation at key residues involved in metal coordination.

OXA-10 differs from OXA-13 and OXA-48 in that the OXA-10 salt bridges require the octahedral coordination of a metal ion to enable dimerization. Comparing the sequence and structure of OXA-66 to that of OXA-10, the hydrophobic interactions that help to promote dimerization are not present in OXA-66 (Fig. 3). Additionally, OXA-10 requires the presence of a divalent metal ion coordinating in an octahedral orientation with E190 and E227 from one chain and H203 from the other alongside several water atoms [20]. Although our structure contains two zinc ions, these are not bound at the expected dimer interact and the arrangement of charges for co-ordinating a metal ion at the dimer interface is not conserved in OXA-66. Instead, a serine, tyrosine and valine are present at this site resulting in disrupted charge interactions suggesting that OXA-66 may not form dimers in the presence of metal ions (Fig. 3). Previously, it was thought that the β−3 and Ω-loops played an important role in the dimerization process [4, 5]. However, on comparing this interface and the salt bridges, we conclude that these reports are unlikely to affect the overall dimerization of the enzyme and instead are more likely to play a mechanistic role. Previous crystal structures of OXA-51 have been crystallised with one or four molecules within an asymmetric unit [3, 18], while our structure contains just one molecule per asymmetric unit which corresponds to Matthews Coefficient of 2.82 and crystal’s solvent content of 56.4 %. In contrast the asymmetric units of dimeric OXAs such as OXA-10, OXA-13 and OXA-48 commonly contain multiple copies of the respective enzymes [19–21]. While not definitive, this suggests that dimerization may be less favoured in the OXA*Ab* enzymes. Further work using appropriate analytical methods such as size exclusion chromatography with multiple angle light scattering (SEC-MALS), or dynamic light scattering (DLS), is needed to determine the true oligomeric state of this group of enzymes.

Overall, we demonstrate that the most prevalent OXA*Ab* enzyme OXA-66 has structural similarity to OXA-51. On the bases of sequence and structural analyses, we also suggest that resides at the dimeric interface suggest the OXA*Ab* enzymes may exist as monomers.

## Supplementary Data

Supplementary material 1
