# Supplementary material for: OXA-66 structure and oligomerisation of OXAAb enzymes
Source: Access Microbiol. 2022 Oct 3;4(10):acmi000412. doi: 10.1099/acmi.0.000412 (PMC9675178; doi:10.1099/acmi.0.000412)

Supplementary Material

Figure S1: Active site of OXA-66 (blue) and the previously solved OXA-51 structure (gold) co-crystallized with Doripenem (green) (PDB ID: 5L2F).

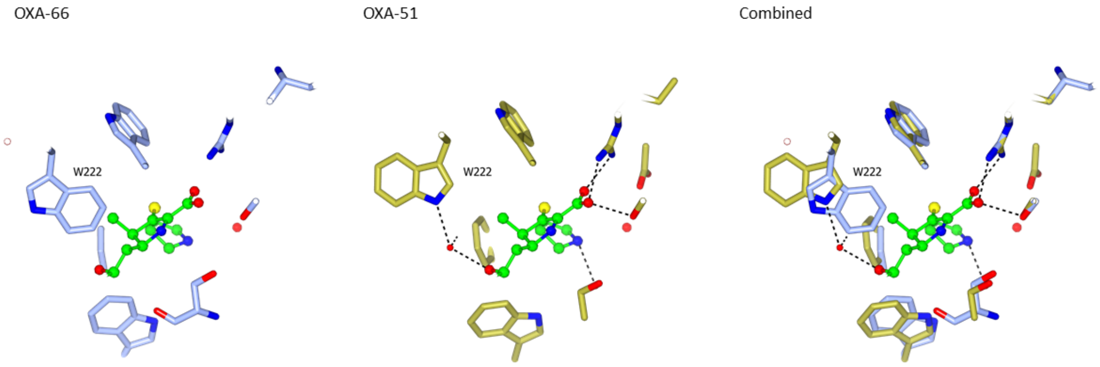

Supplement: Supplementary material 1 [file acmi-4-412-s001.pdf]
